# Tuberculous Enteritis Presenting as Acute Appendicitis and Perirectal Abscess

**DOI:** 10.1155/2018/6068258

**Published:** 2018-02-25

**Authors:** Kelechukwu U. Okoro, Maria Gomez De La Espriella, Douglas J. Grider, Anthony W. Baffoe-Bonnie

**Affiliations:** ^1^Department of Internal Medicine, Virginia Tech Carilion-School of Medicine and Research Institute, Roanoke, VA, USA; ^2^Department of Internal Medicine, Section of Infectious Diseases, Virginia Tech Carilion-School of Medicine and Research Institute, Roanoke, VA, USA; ^3^Department of Pathology, Virginia Tech Carilion-School of Medicine and Research Institute, Roanoke, VA, USA

## Abstract

*Mycobacterium tuberculosis* has a wide variety of presentations. A rare occurrence is gastrointestinal tuberculosis. It may occur anywhere along the alimentary canal but usually occurs in the ileocecum with rare involvement of the appendix.

## 1. Introduction

*Mycobacterium Tuberculosis* (TB) is the second most common infectious cause of death worldwide with humans as the only known reservoir. Approximately one third of the world's population is affected [1]. It may manifest in any organ system and mimic many different diseases. For these reasons, it has been colloquially referred to as the great imitator. The respiratory system is most commonly affected, followed in descending order by the musculoskeletal, genitourinary, reproductive, central nervous, and gastrointestinal systems [2]. When the gastrointestinal (GI) system is involved, the ileocecum is most often affected, with appendicular involvement a rarity [3, 4]. Gastrointestinal TB is usually referred to as tuberculous enteritis.

Tuberculous enteritis had been mostly eradicated in the United States due to improved standard of living, pasteurization of milk, control of bovine tuberculosis, and introduction of antitubercular therapy [4]. More recently, the incidence of TB is slowly rising due to poverty, overcrowded housing, homelessness, drug abuse, inadequate access to health care, increase in migration from countries where TB is endemic, and the HIV epidemic. It is important to also note that patients who develop acquired immunodeficiency syndrome (AIDS) most commonly present with atypical presentations of TB, more virulent disease, and extrapulmonary TB [4, 5]. Other niches for TB proliferation include penitentiaries, nursing homes, and homeless shelters [4]. This case highlights an unusual case of tuberculous enteritis presenting as rectal pain in an immunocompetent male.

## 2. Case Presentation

A 22-year-old Hispanic male who recently emigrated from Guatemala within the last six months, without any previous medical history presented with a chief complaint of rectal and abdominal pain. Onset of the pain was four days before; however, he had been experiencing other symptoms for approximately three months. He reported productive cough, pyrexia, chills, night sweats, fatigue, and weight loss. He denied nausea, vomiting, diarrhea, melena, hematochezia, and rectal manipulation. Vital signs revealed temperature of 99.8°F, blood pressure 105/67 mmHg, pulse 91 bpm, respiratory rate 20 bpm, and oxygen saturation 99%. Labs revealed sodium 136 mEq/L, potassium 4.5 mEq/L, chloride 99 mEq/L, bicarbonate 22 mEq/L, creatinine 0.54, WBC 9.3 k/*μ*l, hemoglobin 11.0 g/dl, and platelet 416 k/*μ*l.

**Figure 1:**
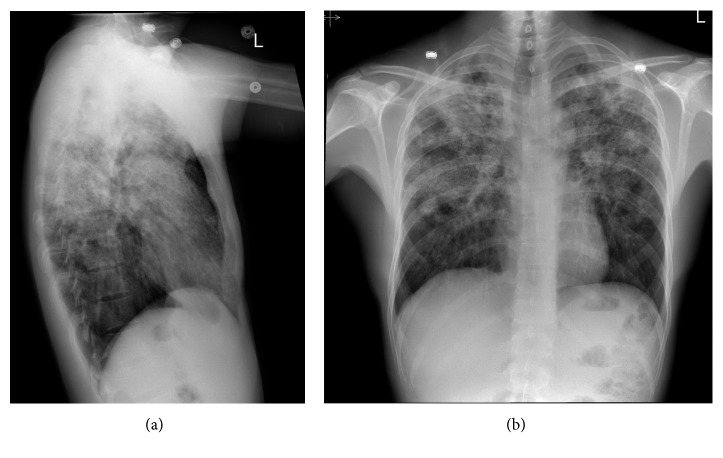
(a, b) PA CXR showing biapical pleural thickening associated with parenchymal scarring, bronchiectasis, nodularity, and superimposed infiltrates.

**Figure 2:**
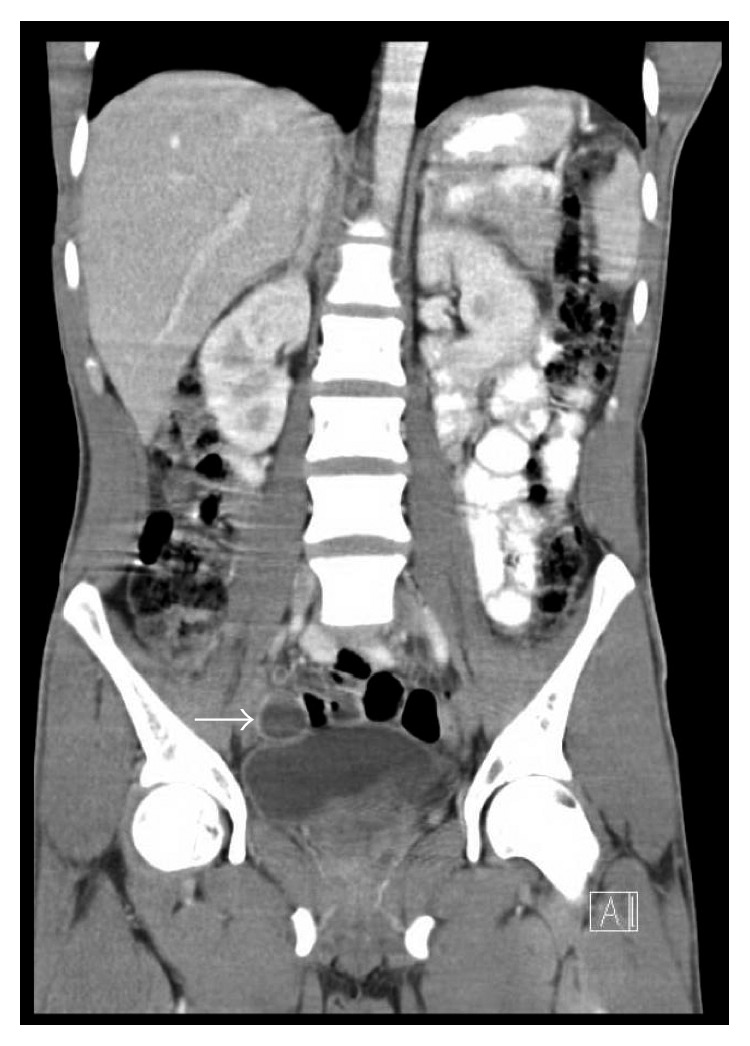
Solid white arrow outlining fluid-filled appendix suggestive of appendicitis.

**Figure 3:**
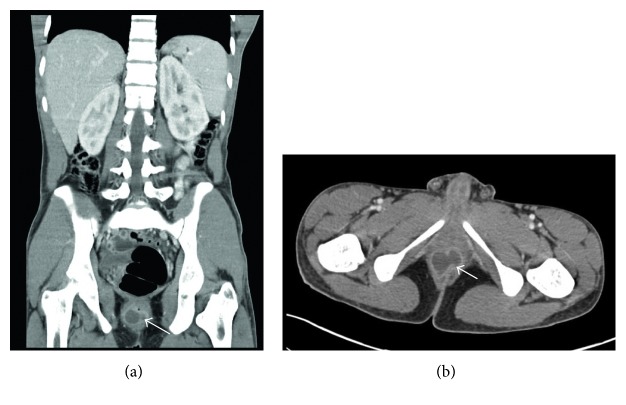
Solid dense arrow delineating perirectal abscess in the saggital (a) and coronal (b) view of the abdominal CT scan.

**Figure 4:**
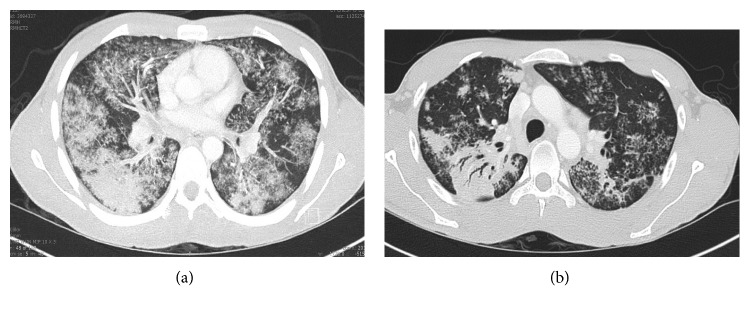
(a) Diffuse airspace patchy consolidation with distention of bronchi and fluffy infiltrates. (b) Dense consolidation in the superior aspect of the right lower lobe with air bronchograms.

On physical examination, there was palpable right lower quadrant tenderness accompanied by abdominal rigidity and involuntary guarding. The patient was not amenable to digital rectal examination due to reported pain. However, illuminated visual examination of the rectum did not reveal any abnormalities. Due to the constellation of travel history, cough, and constitutional symptoms, a chest X-ray (CXR) was performed. It revealed biapical pleural thickening associated with parenchymal scarring, bronchiectasis, nodularity, and superimposed infiltrates (Figure 1). Pulmonary tuberculosis (TB) was highly suspected, and the patient was placed in an isolation room. Further imaging was accomplished with computed tomography (CT) of the abdomen and pelvis with intravenous and oral contrast. This revealed acute appendicitis in the right lower quadrant congruent with earlier physical examination findings (Figure 2). It also revealed a 3.1 × 1.9 cm dumbbell-shaped loculated fluid collection anterior to the anus consistent with perirectal abscess (Figure 3) confirming the etiology of his rectal discomfort. Finally, CT gave more insight into suspected pulmonary TB as it revealed nodular and patchy consolidation in both lung bases along with dense consolidation, fluffy airspace infiltrates, distended, and distorted bronchi (Figure 4).

**Figure 5:**
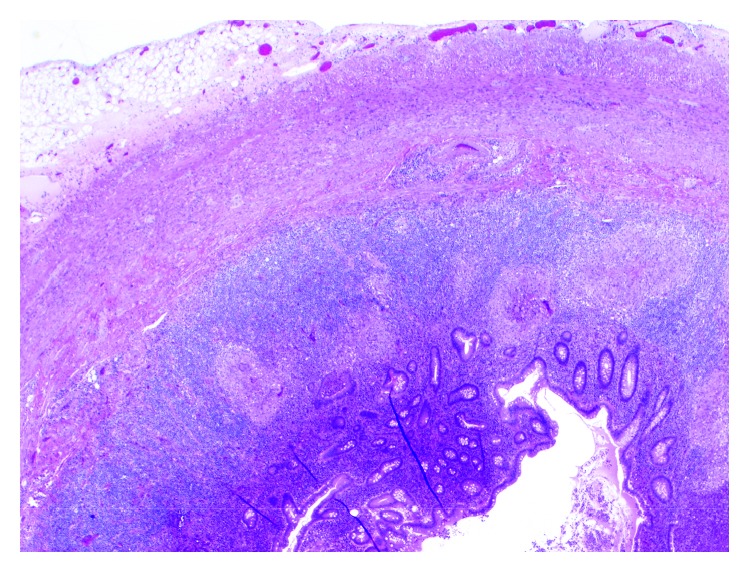
Granulomatous appendix (2x; 20 magnification).

**Figure 6:**
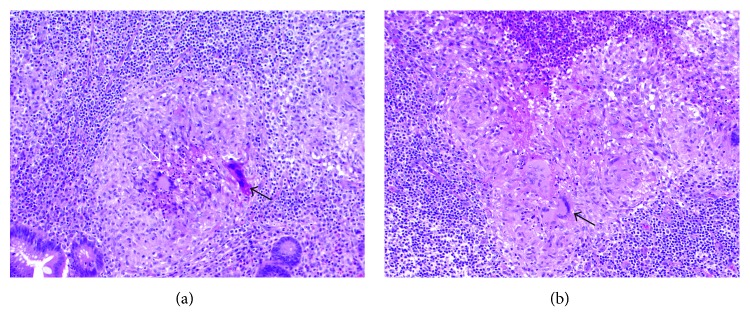
(a, b) H&E stain showing granulomatous inflammation of the appendix with solid black arrows depicting multinucleated giant cells. Central necrosis can be seen in image A outlined by solid white arrow (10x; 100).

**Figure 7:**
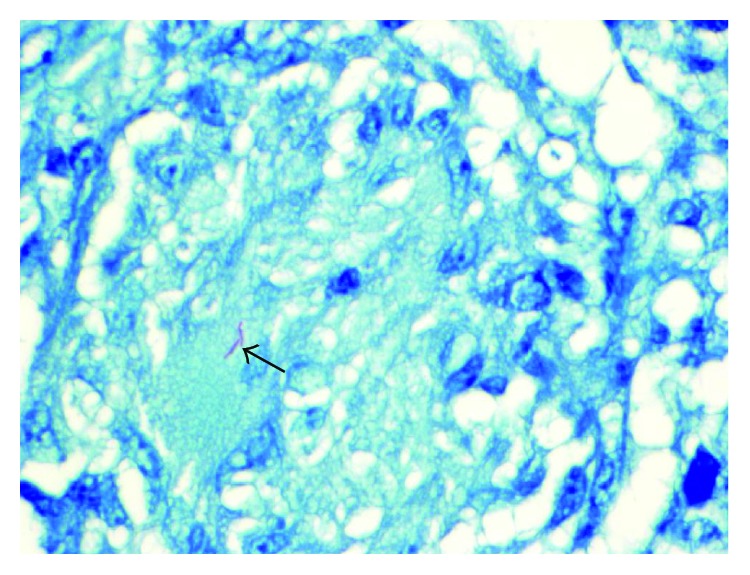
Solid black arrow depicting Ziehl-Neelsen AFB stain positive for acid-fast positive bacilli in the cytoplasm of a multinucleated giant cell (60x; 600).

The patient was taken to the operating room expeditiously where he underwent diagnostic bronchoscopy with bronchoalveolar lavage (BAL), laparoscopic appendectomy, and incision and drainage of the perirectal abscess. By postoperative day one, cultures from BAL, sputum, and perirectal abscess were positive for acid-fast bacilli (AFB). Cultures from the perirectal abscess were also positive for beta-hemolytic group C streptococcus. Gross pathology of the appendix revealed a dusky, congested, red-brown serosa. When sectioned, we appreciated a dilated lumen measuring approximately 0.5 cm in diameter and an appendiceal wall thickness averaging at 0.3 cm. The mucosa was noted to be tan-pink, glistening, and congested. The lumen of the appendix contained abundant gray-pink semisolid contents. A discrete perforation was not appreciated. Histologic analysis revealed a focally effaced mucosa albeit where present, the epithelium was without atypia. Foci of acute and granulomatous inflammation were appreciated (Figure 5) along with epithelioid histiocytes and multinucleated giant cells (Figure 6). Initial acid-fast staining was negative, but a repeat stain revealed the presence of acid-fast bacilli in the cytoplasm of a multinucleated giant cell (Figure 7).

The patient was started on antitubercular drugs that included a combination of rifampin, isoniazid, pyrazinamide, and ethambutol. Based on sensitivities, he was also started on antibiotics for management of the streptococcal infection. The patient did well postoperatively and tolerated both antibacterial and antitubercular treatments without complication.

## 3. Discussion

Tuberculous enteritis may occur at any age. It does not exhibit any sexual preponderance, occurring equally in both male and female populations. Symptoms vary depending on the pathogenesis. There are four main mechanisms which may lead to tuberculous enteritis. These include swallowing of infected sputum in active pulmonary tuberculosis, ingestion of contagious milk from cattle infected with bovine TB, direct extension from adjacent organs, hematogenous spread from active pulmonary TB, miliary TB, or silent bacteremia during the primary phase of TB [4–6]. After the bacillus enters the gastrointestinal tract, it traverses the mucosa to lodge in the submucosa leading to inflammatory changes including cellular infiltration, lymphatic hyperplasia, serosal, and submucosal edema [4]. The eventual result of inflammation is production of granuloma which causes small papillary mucosal elevations, lymphangitis, endarteritis, and fibrosis. In due course mucosal ulceration develops along with caseating necrosis and narrowing of the intestinal lumen [4]. The lesions that occur are categorized grossly as ulcerative, hypertrophic, ulcerohypertrophic, and fibrotic stricture [6]. Ulcerative lesions are most commonly seen in the small intestine while hypertrophic and ulcerohypertrophic lesions mostly occur in the ileocecal region. The alimentary tract is affected 50% of the time versus the peritoneum which is involved 43% of the time, with the mesenteric lymph nodes exhibiting an 8% incidence [4]. In the GI tract, the ileocecum is mostly affected followed by jejunoileum, colon, anorectum, stomach, esophagus, duodenum, and appendix.

Patients may present with acute, subacute, or chronic symptoms. More commonly patients present with subacute to chronic complaints with symptoms onset varying from one month to one year. Approximately 20% of patients have symptoms for a month or less at the time of presentation [6]. If patients present with acute symptoms, it is usually secondary to acute intestinal obstruction, acute peritonitis with or without perforation, acute mesenteric lymphadenitis, or acute appendicitis [7]. The symptoms and signs are nonspecific. In this case, the patient presented to the emergency department with a chief complaint of rectal pain. Through review of systems, pulmonary disease was suspected prompting a CXR. A thorough history revealed abdominal pain which was corroborated by physical examination findings. A high index of suspicious prompted an abdominal CT revealing disease that was eventually confirmed by gross tissue examination and histopathology. The inflamed appendix showed epithelioid granulomatous inflammation with necrosis on microscopy (Figures 5 and 6), and confirmatory acid-fast staining revealed positive bacilli (Figure 7).

The most common presenting symptom is abdominal pain which occurs in approximately 85%–93% of patients [4, 6]. Abdominal tenderness to palpation usually accompanies reports of pain, and a mass may be palpated in the right lower quadrant (RLQ) in 25–50% of patients. The etiology of RLQ abdominal mass includes hyperplastic cecal tuberculosis, tubercular lymphadenitis, and rolled-up omentum [8]. It is important to note that the location of pain may also vary with the site of the lesion. Another 66% of patients experience weight loss while pyrexia occurs in 35–50%, and diarrhea in 20% of patients [6].

The most common complication of tuberculous enteritis is obstruction which may be acute in onset but more frequently presents as partial obstruction. This may evolve to a complete obstruction if the disease remains untreated [6, 8]. The small bowel is affected more frequently than the colon. Mechanisms for obstruction include inflammatory thickening of the bowel wall secondary to fibrotic contraction from healing ulcers and kinking or constriction of the intestine from adhesions or lymphadenopathy [8]. Other complications include bowel perforation, fistulization, malabsorption, and intestinal hemorrhage.

Diagnosing tuberculous enteritis early is difficult because of the myriad of ways it can present. Thus, a high index of suspicion is required. In many instances, patients are misdiagnosed with somewhat similar presenting diseases including inflammatory bowel disease or infectious agents such as *Yersinia*, *Campylobacter*, or Histoplasma. This is due to the fact that gross examination of tuberculous enteritis may reveal pancolonic inflammation or increased mesenteric fat associated with caseating adenopathy similar to inflammatory bowel disease and other aforementioned lesions that may lead to misdiagnosis [4]. Once TB infection is suspected, imaging techniques such as CT or MRI should be considered. Also on endoscopic examination, biopsy samples can be collected rather than proceeding to more invasive open laparotomy or laparoscopy. Treatment is usually accomplished by a combination of antitubercular medications with surgical intervention required only when complications develop.

## 4. Conclusion

Gastrointestinal tuberculosis is a rare disease but not as uncommon as thought to be. A high index of suspicion is requisite when examining patients whose clinical presentation mirrors the differential diagnoses of gastrointestinal tuberculosis.
